# Supplementary material for: Table tennis coaching system based on a multimodal large language model with a table tennis knowledge base
Source: PLoS One. 2025 Feb 13;20(2):e0317839. doi: 10.1371/journal.pone.0317839 (PMC11824982; doi:10.1371/journal.pone.0317839)
Supplement: S1 Appendix — (PDF) [file pone.0317839.s001.pdf]

AI乒乓教练系统详细评估问卷

请观看五个新手乒乓球训练中的失误视频，对AI乒乓教练系统提供的失误分析做出评价。

指导准确性：

1. 您认为系统分析的技术动作时的准确度如何？
   - [ ] 非常不准确
   - [ ] 有点不准确
   - [ ] 一般
   - [ ] 准确
   - [ ] 非常准确
2. 系统在识别非受迫性失误方面表现如何？
   - [ ] 非常不准确
   - [ ] 有点不准确
   - [ ] 一般
   - [ ] 准确
   - [ ] 非常准确
3. 系统对比赛风格和技术特点的理解程度如何？
   - [ ] 完全不理解
   - [ ] 理解较差
   - [ ] 一般
   - [ ] 较好理解
   - [ ] 非常理解

实用性：

4. 系统提供的建议在实战中可能的应用效果如何？
   - [ ] 完全无效
   - [ ] 效果一般
   - [ ] 有一定效果
   - [ ] 效果显著
   - [ ] 非常有效
5. 系统的建议是否及时并且便于执行？

- [ ] 非常不及时
- [ ] 有点不及时
- [ ] 一般
- [ ] 及时
- [ ] 非常及时

个性化程度：

6. 系统是否能根据个人技术特点提供定制化建议？

- [ ] 完全没有
- [ ] 较少
- [ ] 一般
- [ ] 较多
- [ ] 非常多

7. 系统在调整训练内容和难度上是否考虑了个人需求？

- [ ] 完全没有
- [ ] 较少
- [ ] 一般
- [ ] 较多
- [ ] 非常多

可理解性：

8. 系统提供的技术建议是否易于理解？

- [ ] 非常难以理解
- [ ] 有点难以理解
- [ ] 一般
- [ ] 容易理解
- [ ] 非常容易理解

9. 系统的用户界面（UI）是否直观易用？

- [ ] 非常不直观
- [ ] 不够直观
- [ ] 一般
- [ ] 较为直观
- [ ] 非常直观

10. 系统的反馈和指导是否具有实用性，能否从中获取有价值的信息？

- [ ] 完全没有价值
- [ ] 价值不大
- [ ] 有一定价值
- [ ] 很有价值
- [ ] 非常有价值

额外建议：

11. 您有什么其他建议或想要分享的体验吗？

    [开放性回答]
