# Supplementary material for: Table tennis coaching system based on a multimodal large language model with a table tennis knowledge base
Source: PLoS One. 2025 Feb 13;20(2):e0317839. doi: 10.1371/journal.pone.0317839 (PMC11824982; doi:10.1371/journal.pone.0317839)
Supplement: Attachment — Submitted filename: PONE-D-24-31115.docx [file pone.0317839.s002.docx]

PONE-D-24-31115

**GENERAL CONCEPT COMMENTS**

Congratulations to the authors for their diligent work and valuable contribution to the field through this research endeavour. After carefully reviewing the article and considering the recommendations provided, it is evident that the manuscript presents valuable insights into the topic. The article “Table Tennis Coaching System Based on a Multimodal Large Language Model with a Table Tennis Knowledge Base” is an innovative and promising contribution to the intersection of sports and AI. The application of multimodal large language models (LLMs) for table tennis coaching has potential to revolutionize training by providing personalized, precise, and cost-effective guidance. However, there are several areas in which the article can be improved to meet the standards of scientific rigor typically applied in research, particularly in the methods section, which lacks crucial details.

**SPECIFIC COMMENTS**

1. **INTRODUCTION**

Specific textual recommendations for accuracy and precision:

(P. 8) - The introduction provides a good foundation on the benefits of table tennis, but the transition to AI application feels abrupt. A stronger link between the issues with current table tennis training methodologies and how AI offers an innovative solution would improve the flow.

•	Suggestion: Expand on why current technologies (like VR or traditional video analysis) are insufficient for personalized, high-efficiency training and how multimodal LLMs can bridge this gap.

(P. 8) – Correct 10 and 11 reference formats.

(P. 8) – Correct 12 and 13 reference formats.

(P. 8) - “Children who regularly engage in table tennis training demonstrate better physical composition and fitness, compared to active children who do not participate in regular physical activities[3]” The sentence seems confusing; consider revising it for clarity by specifying the comparison group and the benefits more clearly.

(P. 8) – The acronym “AI” appears before it is introduced in the text. To improve clarity, “Artificial Intelligence (AI)” should be introduced the first time it’s mentioned.

(P. 9) – Correct 20 and 21 reference formats.

(P. 9) – Correct 23 and 24 reference formats.

(P. 9) - The discussion of technological advancements (VR, AI) could benefit from a more structured comparison of existing tools used in sports and how this system differs or improves upon them.

2. **METHODS**

Consider revising the text to avoid using first-person language, as scientific writing typically favors a more objective tone. For example, instead of “I positioned my smartphone,” use “The smartphone was positioned” to maintain formality and focus on the research process.

Specific textual recommendations for accuracy and precision:

(P. 9) - The method involving the placement of a single smartphone for capturing video is innovative for its simplicity. However, it may limit the system’s ability to capture complex footwork and body dynamics.

•	Suggestion: Acknowledge that using only one camera may reduce the accuracy of certain movements (like footwork) and propose future improvements, such as adding multiple camera angles. This limitation should be made clear in the methodology section as well as in the limitations section.

(P. 10) – The figure 1, 2, 3, 4 must appear immediately after its introduction in the text.

(P. 10) - While the system mentions GPT-4 as the core model for providing training recommendations, there is insufficient detail about how the model was trained or adjusted for this specific application. The use of LLMs in a complex task such as table tennis coaching demands more transparency, especially around: (i) Whether the model was pre-trained or adjusted; (ii) The dataset used for fine-tuning (e.g., how much data, and from which sources?); (iii) Hyperparameters such as learning rate, batch size, and optimization algorithms used in training, similar to how traditional statistical methods require reporting of confidence intervals or normality checks; (iv) Was any data augmentation or preprocessing applied to enhance the model’s performance?.

(P. 11) - The integration of motion capture data (from OpenPose) and visual recognition data into the LLM is a significant aspect of the system, but there is no clear explanation of how this data is formatted or fed into GPT-4. The authors must explain how multimodal data is handled, much like a traditional study would report assumptions checks or data transformations before analysis.

Additional questions also rise: (i) Are the visual and motion data converted to text or encoded in another form before being processed by the model?; (ii) If the data is fed into the model as structured inputs, provide details on the data pipeline and preprocessing steps; (iii) Without this information, readers are left uncertain about the complexity of the integration between the multimodal data and the LLM.

(P. 11) - OpenPose is a robust tool for motion capture, but it is important to note that its performance may degrade in cluttered or poorly-lit environments, which could affect amateur users.

•	Suggestion: Add a section on the environmental limitations of OpenPose and how it affects data accuracy. Including some recommendations on ideal lighting and camera quality for users could be helpful.

(P. 11) - The core functionality of the system relies on GPT-4 generating recommendations based on visual and motion data inputs. However, there is no mention of the prompts, or the structure of the input data used to query the language model. This is a critical detail for understanding how the model generates athlete-specific advice. The article should provide examples of the exact prompts given to GPT-4, much like one would report the mathematical formulas or procedures used in a statistical test. For instance: (i) What input does the model receive? Is it a natural language description, a set of structured variables, or something else?; (ii) Are there any predefined templates or rules for these prompts?; (iii) This omission weakens the study’s transparency, as it is crucial to understand the interaction between multimodal data and the LLM.

(P. 11) - The study should also address potential concerns about overfitting, particularly because it is difficult for AI models to generalize across varied real-world scenarios without extensive data. It’s important to understand if the system was tested on diverse datasets, akin to how normality or homogeneity tests are used in statistical research. In example: (i) Was the model validated on external datasets or players not included in the original training data? And (ii) Did the authors apply regularization techniques (e.g., dropout layers, early stopping) to prevent overfitting?

(P. 12) - The dataset is well-defined in terms of error categorization (e.g., wrist errors, footwork errors), but the process of how data were annotated by experts could be more detailed.

•	Suggestion: Include details on the inter-rater reliability among the coaches who annotated the errors. How consistent were they in identifying these errors? A mention of this would improve the perceived robustness of the dataset.

(P. 12 L. 5–25) - The system’s accuracy in identifying table tennis errors is reported, but crucial details, like confidence intervals or variance in accuracy across different datasets, are missing. Just as traditional statistical analyses require detailed metrics (effect sizes, p-values), AI models must be rigorously evaluated using standard performance metrics. In example: (i) Report accuracy, precision, recall, and F1-scores for error identification; (ii) Include confidence intervals or standard deviations for these metrics to indicate the model’s robustness; (iii) Clarify whether cross-validation was used to ensure that the model is not overfitting to a particular set of data; and (iv) Include a confusion matrix to show where the model succeeds or fails in classifying various errors (e.g., racket position vs. footwork).

(P. 12 L. 10–20) - The article does not clarify whether the outputs generated by GPT-4 were evaluated for quality, accuracy, or consistency. This is essential, as one would expect an evaluation of any AI system’s output similar to how confidence intervals or p-values are reported in statistical studies. As a reviewer I have a few questions: (i) Was the quality of GPT-4’s recommendations assessed? Were there metrics or cross-validation techniques applied to ensure the model consistently provides correct or useful feedback?; (ii) Did the authors perform any error analysis to understand common mistakes made by the model (e.g., false positives or incorrect classifications)?; (iii) If experts evaluated the system, what were the specific criteria used to measure GPT-4’s performance?

(P. 12) – Table 1 should be introduced in the text before the actual table.

3. **RESULTS**

Specific textual recommendations for accuracy and precision:

(P. 13) – Figure 5 should be introduced in the text prior to its appearance in the document.

(P. 13) – The figure 6 must appear immediately after its introduction in the text.

(P. 13) - The system demonstrates impressive accuracies (73% for arm-related errors and 82% for racket errors). However, these percentages are not sufficient for real-world use if aiming for higher levels of training (beyond beginner-level players).

•	Suggestion: Clarify that these accuracy metrics are primarily for beginners and that further refinement is needed for advanced players. Also, suggest ways to increase accuracy, such as incorporating biomechanics data or higher-resolution tracking.

(P. 14) - The comparison between GPT-4, Gemini Pro, and Qwen-VL is useful, but the decision criteria for selecting GPT-4 should be elaborated.

•	Suggestion: Add more detailed metrics to the model comparison (e.g., computation time, resource consumption, scalability) and explain why GPT-4 was chosen over the others in those dimensions, not just for accuracy and false positives.

(P. 14) – Figure 7 should be introduced in the text prior to its appearance in the document.

(P. 15) – Figure 9 should be introduced in the text prior to its appearance in the document.

4. **DISCUSSION**

Specific textual recommendations for accuracy and precision:

(P. 16) - The cost analysis focuses primarily on beginner-level applications, but there is no mention of scaling this system for more advanced or professional users.

•	Suggestion: Include a section that discusses how the system could be adapted for semi-professional or professional players, with potentially more advanced (and more expensive) hardware. This would broaden the scope of the application and make the study more impactful.

(P. 16) - The lower accuracy in recognizing footwork errors is mentioned, but more detail should be given regarding how this will be improved in future research.

•	Suggestion: Propose specific technical solutions, such as integrating a multi-camera system or combining inertial measurement units (IMUs) with visual data. Without a clear plan, it may appear that this limitation will remain unaddressed.

5. **CONCLUSION**

Specific textual recommendations for accuracy and precision:

(Page 17) - The conclusion touches on future improvements in footwork recognition and training for high-level athletes, but these should be expanded with more concrete plans.

6. **REFERENCES**

In most of the references, the symbol ‘[J]’ appears. Please confirm whether this is intentional.
