# Supplementary material for: Table tennis coaching system based on a multimodal large language model with a table tennis knowledge base
Source: PLoS One. 2025 Feb 13;20(2):e0317839. doi: 10.1371/journal.pone.0317839 (PMC11824982; doi:10.1371/journal.pone.0317839)
Supplement: Attachment — Submitted filename: Response to Reviewers.docx [file pone.0317839.s003.docx]

Dear Editors of PLOS ONE,

Greetings!

We sincerely thank you for your thorough review of our manuscript and for providing detailed publication recommendations. We have carefully addressed each of the points raised and have made the necessary revisions to our submission. Below, we outline our responses to each requirement:

1. **Manuscript Style and File Naming**

We have ensured that our manuscript adheres to PLOS ONE's style requirements, including the correct file naming conventions. All sections of the manuscript have been adjusted accordingly to meet these guidelines.

2. **Code Sharing Compliance**

The de-identified raw data used in this study will be made available in a public online repository following publication, Preprocessing and analysis code has been made available in a public GitHub repository:

https://github.com/mwlsus/ttcs_by_llm

This ensures that our code follows best practices and facilitates reproducibility and reuse of our research.

3. **Ethical Approval Clarification**

Ethical approval

Dear Editor：

The focus of this research is on the development of an AI-based table tennis coaching system. This system primarily involves the use of **motion capture, computer vision, and AI-based data analysis** to improve the accuracy and effectiveness of table tennis training. The study employs **visual recognition technology** to analyze ball trajectories and identify common technical errors among beginners.

Specifically:

1. **Non-identifiable Data Collection**: Data are anonymized and involve only **technical and movement data** (e.g., ball trajectory, racket positioning, and limb motions) captured through visual recognition technologies. No sensitive or personal identifiers are involved.
2. **Minimal Risk**: The study's focus on low-risk data collection and the use of **standard sports monitoring equipment** (such as a 1080p camera on a tripod) reduces the possibility of risk to participants. Additionally, the captured data solely include general movements rather than individual-specific biometric information**Public Settings and Full Consent**: Participants were briefed about the study's purpose and scope, with **informed consent obtained** before data collection. Data protection measures, such as **encryption and anonymization**, further support privacy compliance.
3. A total of 10 table tennis experts were selected to evaluate thepotential of the AI-based table tennis coaching system. The survey was primarily distributed through online platforms such as WeChat and Wenjuanxing. All participants were adults (18 years or older). Participants filled out the questionnaire by clicking through it continuously. Before completing the questionnaire, they were informed of the purpose of the survey and were notified that submitting the questionnaire would be considered as informed consent. Participants could exit the questionnaire at any time during the process.

Given the nature of this data (non-sensitive, anonymized, and low-risk) and the fact that it does not involve intrusive data collection, this study aligns with the institution's policy of not requiring formal ethical review. The research emphasizes **technical advancements** in AI coaching systems rather than personal data analysis, thus maintaining ethical standards without needing specific ethics committee approval.Because my experiment started at Beijing Sport University, I have obtained the stamp from the Ethics Review Committee of Beijing Sport University.

Yours sincerely，

Wenlong Ma

November 8, 2024

4. **Competing Interests Statement**

We have updated the Competing Interests section in the online submission form to state: "The authors have declared that no competing interests exist.". This statement is also included in our cover letter for your reference.

5. **Data Availability**

Following PLOS ONE's data availability policy, all data underpinning our findings have been deposited in the Github repository:

<https://github.com/mwlsus/ttcs_by_llm>

We have updated the manuscript to include these links. Additionally, we have ensured that no data breaches ethical or legal standards, and any sensitive information has been appropriately anonymized.

6. **Author Information Addition**

Wenlong Ma^1^,（The first author）

Yang Liu^2^,

Qing Yi^3^,

Xutao Liu^4^,

Wei Xing^5^,

Rongji Zhao ^6^ ,

Huan Liu ^7^ ,

Rongzhi Li^1*^（Corresponding author）

7. **Consent for Publication of Images**

For Figures 2 and 3，I have already included the revision in the introduction to the methods section manuscript."The revision is as follows：

For Figures 2 and 3,the individuals in this research have provided written informed consent (as outlined in the PLOS consent form) for the publication of these images.

For Figure6 I have already included the revision in the 3.2 Guidance for Unforced Errors in Table Tennis Novices in the manuscript The revision is as follows：

the individuals in this research have provided written informed consent (as outlined in the PLOS consent form) for the publication of these images.

8. **Table Reference in Manuscript**

We have reviewed the manuscript and ensured that Table 1 is appropriately referenced within the text. Relevant sections now include explicit mentions of Table 1 to guide readers effectively.

Once again, we appreciate the constructive feedback and guidance provided by the PLOS ONE editorial team. We believe that these revisions have strengthened our manuscript, and we look forward to your favorable consideration.

**Response to Reviewer1**

**Response to GENERAL CONCEPT COMMENTS：**Congratulations to the authors for their diligent work and valuable contribution to the field through this research endeavour. After carefully reviewing the article and considering the recommendations provided, it is evident that the manuscript presents valuable insights into the topic. The article “Table Tennis Coaching System Based on a Multimodal Large Language Model with a Table Tennis Knowledge Base” is an innovative and promising contribution to the intersection of sports and AI. The application of multimodal large language models (LLMs) for table tennis coaching has potential to revolutionize training by providing personalized, precise, and cost-effective guidance. However, there are several areas in which the article can be improved to meet the standards of scientific rigor typically applied in research, particularly in the methods section, which lacks crucial details.
**Response：**Thank you for your insightful and constructive feedback on our article. We truly appreciate your recognition of our efforts and the potential impact of our research on AI-driven table tennis coaching systems. Your comments regarding the innovative nature of applying multimodal large language models in this domain are very encouraging.We acknowledge the need for more detail in the methods section and will carefully address this to enhance clarity and replicability. Your feedback highlights key areas for improvement, and we are committed to refining our manuscript to meet the highest standards of scientific rigor. Thank you once again for your valuable guidance.

**1.Introduction：**

**Response to comments: 1.**Specific textual recommendations for accuracy and precision:(P. 8) - The introduction provides a good foundation on the benefits of table tennis, but the transition to AI application feels abrupt. A stronger link between the issues with current table tennis training methodologies and how AI offers an innovative solution would improve the flow.Suggestion: Expand on why current technologies (like VR or traditional video analysis) are insufficient for personalized, high-efficiency training and how multimodal LLMs can bridge this gap.

**Response：**I introduced the numerous benefits of table tennis. Following this, I wrote,Interest in learning table tennis can inspire individuals to be more willing to participate in the sport, thereby demonstrating the numerous benefits of playing table tennis，and point out an effective and scientifically-based table tennis training method can stimulate table tennis learners' interest.However, the current table tennis training methods are relatively limited, still adhering to a teacher-centered approach where instructors lecture while students listen. Additionally, teachers predominantly follow traditional textbook-based teaching methods, which lack personalized instruction and place students in a passive learning state, inevitably affecting their interest in learning table tennis skills.The evolution of technology has markedly advanced table tennis training methods, integrating various technological tools to augment training efficiency and outcomes[6]-7]. The application of Virtual Reality (VR) technology enables learners to train within simulated environments[8], which not only heightens learner engagement but also allows for specialized skill practice unaffected by external conditions[9]-10].However, VR technology exhibits significant limitations in delivering precise and personalized guidance. VR training environments primarily emphasize scene recreation and often lack the capability to dynamically adjust based on real-time feedback from learners. As a result, VR are unable to provide targeted recommendations for technical improvement. While the benefits of VR technology have been introduced earlier, here, we turn to highlight its limitations, specifically its inability to provide personalized training suggestions and plans, and why this occurs. This then leads to the connection with AI applications and introduces what AI is.In recent years，AI has made significant advancements，it can provide personalized training recommendations and plans for beginners, strengthening their understanding of technical movement learning. Moreover, it lays a solid foundation for beginners to enhance their proficiency in technical skills.Artificial intelligence is the science of endowing programs with the ability to change themselves for the better as a result of their own experiences[11],which sets the stage for the introduction of multimodal large language models in the following section.

**Response to comments: 2.**Children who regularly engage in table tennis training demonstrate better physical composition and fitness, compared to active children who do not participate in regular physical activities[3]” The sentence seems confusing; consider revising it for clarity by specifying the comparison group and the benefits more clearly.
**Response：**Table tennis players show better cardiovascular endurance, muscle strength, and flexibility compared to physically peers not engaged in regular sports training[3].

**Response to comments: 3.**The acronym “AI” appears before it is introduced in the text. To improve clarity, “Artificial Intelligence (AI)” should be introduced the first time it’s mentioned.

**Response：**Artificial intelligence is the science of endowing programs with the ability to change themselves for the better as a result of their own experiences[11].

**Response to comments: 4.**The discussion of technological advancements (VR, AI) could benefit from a more structured comparison of existing tools used in sports and how this system differs or improves upon them.

**Response：**Comparison of technological advances: Thank you for the suggestion. We will structure the comparison of current sports tools more clearly and further explain the unique aspects and improvements of the proposed system. In the text, the differences of the AI Table Tennis Coaching System have already been described in detail.This system effectively identifies common errors among beginners and offers targeted guidance and training recommendations. By compiling and analyzing extensive data on table tennis techniques, this study has constructed a comprehensive dataset that supports further research and enhances the AI system’s capabilities. AI table tennis coaching system significantly improves training efficiency and quality while substantially reducing coaching costs.
**2. METHODS**

**Response to comments: 1.**Consider revising the text to avoid using first-person language, as scientific writing typically favors a more objective tone. For example, instead of “I positioned my smartphone,” use “The smartphone was positioned” to maintain formality and focus on the research process.

**Response：**Thank you for your feedback! I appreciate the suggestion to adopt a more objective tone. I’ll revise the text to eliminate first-person language, ensuring the research is presented with clarity and formality.A smartphone was positioned on a tripod to capture clear angles of beginners' forehand, backhand, and serving techniques.
**Response to comments: 2.**(P. 9) - The method involving the placement of a single smartphone for capturing video is innovative for its simplicity. However, it may limit the system’s ability to capture complex footwork and body dynamics.• Suggestion: Acknowledge that using only one camera may reduce the accuracy of certain movements (like footwork) and propose future improvements, such as adding multiple camera angles. This limitation should be made clear in the methodology section as well as in the limitations section.

**Response：**Thank you for your thoughtful insights.I added sentences about using a single camera for motion capture in the introduction section of the methodology."While using a single camera offers simplicity, it may reduce the accuracy in capturing certain movements, particularly footwork. Future improvements could involve the addition of multiple camera angles to enhance precision in detecting footwork and other complex body dynamics.
**Response to comments: 3.**(P. 10) – The figure 1, 2, 3, 4 must appear immediately after its introduction in the text.

**Response：**Thank you for your comment! I will ensure that Figures 1, 2, 3, and 4 are placed immediately after their respective introductions in the text for better clarity and flow.
**Response to comments: 4.**(P. 10) - While the system mentions GPT-4 as the core model for providing training recommendations, there is insufficient detail about how the model was trained or adjusted for this specific application. The use of LLMs in a complex task such as table tennis coaching demands more transparency, especially around: (i) Whether the model was pre-trained or adjusted; (ii) The dataset used for fine-tuning (e.g., how much data, and from which sources?); (iii) Hyperparameters such as learning rate, batch size, and optimization algorithms used in training, similar to how traditional statistical methods require reporting of confidence intervals or normality checks; (iv) Was any data augmentation or preprocessing applied to enhance the model’s performance?.

**Response:** Thank you for your valuable feedback, which has helped us improve our manuscript. Our model is based on GPT-4V and achieves recognition and analysis of unforced errors in table tennis through the collaboration of multimodal input, prompt engineering, and a knowledge base. Since the GPT model has already acquired knowledge about human motion and sports theory during training, our table tennis coach model can effectively guide beginners by activating and integrating the pre-trained prior knowledge of the GPT model using multimodal input, prompt engineering, and a knowledge base. To demonstrate the superiority of this pipeline, we have added a comparative analysis in Section 3.1, illustrating the prediction accuracy of table tennis errors by GPT without a knowledge base and preprocessing, compared to our AI table tennis coach system.
**Response to comments: 5.**(P. 11) - The integration of motion capture data (from OpenPose) and visual recognition data into the LLM is a significant aspect of the system, but there is no clear explanation of how this data is formatted or fed into GPT-4. The authors must explain how multimodal data is handled, much like a traditional study would report assumptions checks or data transformations before analysis.Additional questions also rise: (i) Are the visual and motion data converted to text or encoded in another form before being processed by the model?; (ii) If the data is fed into the model as structured inputs, provide details on the data pipeline and preprocessing steps; (iii) Without this information, readers are left uncertain about the complexity of the integration between the multimodal data and the LLM.

**Response:** We have incorporated the information related to the identification of different errors into Table 2 and highlighted it in the text. The multimodal image data input includes immediate frames of missed shots and motion status diagrams analyzed via OpenPose. These image data are encoded in the standard base64 format for multimodal large models. Combined with prompts and the knowledge base, they are submitted as API-recognizable JSON serialization to the LLM server.We sincerely appreciate your insightful feedback, which has greatly contributed to enhancing the clarity and quality of our manuscript.
**Response to comments: 6.**(P. 11) - OpenPose is a robust tool for motion capture, but it is important to note that its performance may degrade in cluttered or poorly-lit environments, which could affect amateur users.•Suggestion: Add a section on the environmental limitations of OpenPose and how it affects data accuracy. Including some recommendations on ideal lighting and camera quality for users could be helpful.
**Response:** Thank you for your insightful feedback. OpenPose is an exceptionally powerful tool for human motion recognition and capture. We have added content to the manuscript to share the specific configuration we used with OpenPose, including details on camera positioning, resolution, and other relevant parameters.

**Response to comments: 7.**(P. 11) - The core functionality of the system relies on GPT-4 generating recommendations based on visual and motion data inputs. However, there is no mention of the prompts, or the structure of the input data used to query the language model. This is a critical detail for understanding how the model generates athlete-specific advice. The article should provide examples of the exact prompts given to GPT-4, much like one would report the mathematical formulas or procedures used in a statistical test. For instance: (i) What input does the model receive? Is it a natural language description, a set of structured variables, or something else?; (ii) Are there any predefined templates or rules for these prompts?; (iii) This omission weakens the study’s transparency, as it is crucial to understand the interaction between multimodal data and the LLM.

**Response:** We sincerely appreciate the reviewer’s insightful comments. Providing details about our model's input format and prompts is indeed a crucial aspect of our research. Our model utilizes a simplified CO-STAR framework for input prompts, which includes Context, Outcome, Scale, Actor, and Resources. We have highlighted this in Section 2.5 through use case diagrams and descriptions. Thank you once again for your valuable feedback.
**Response to comments:8.**(P. 11) - The study should also address potential concerns about overfitting, particularly because it is difficult for AI models to generalize across varied real-world scenarios without extensive data. It’s important to understand if the system was tested on diverse datasets, akin to how normality or homogeneity tests are used in statistical research. In example: (i) Was the model validated on external datasets or players not included in the original training data? And (ii) Did the authors apply regularization techniques (e.g., dropout layers, early stopping) to prevent overfitting?
**Response:**Thank you for your insightful feedback. Throughout the development of the AI Table Tennis Coach system, the GPT-4V model was not pre-exposed to any content from our dataset, ensuring that the large language model does not deliberately fit the training set. Our AI Table Tennis Coach model focuses solely on identifying and advising on unforced errors during novice training matches. Currently, datasets in this field remain scarce, apart from our work. We are committed to continuing our contributions to the field of table tennis education in the future. In Section 2.5 of the Methods, we clarify that our approach does not intentionally fit the data, and we have included a discussion on the future cross-scenario generalization capabilities of the model.

**Response to comments:9.**(P. 12) - The dataset is well-defined in terms of error categorization (e.g., wrist errors, footwork errors), but the process of how data were annotated by experts could be more detailed.• Suggestion: Include details on the inter-rater reliability among the coaches who annotated the errors. How consistent were they in identifying these errors? A mention of this would improve the perceived robustness of the dataset.
**Response:**Thank you for your insightful feedback. It is indeed essential to provide a detailed explanation of the dataset. We have included Fleiss' Kappa data regarding annotation errors in Table 1 to demonstrate the robustness of the dataset. Additionally, we have elaborated on the process of expert annotation. These enhancements aim to clarify and facilitate a better understanding of our dataset's composition.

**Response to comments:10.**(P. 12 L. 5–25) - The system’s accuracy in identifying table tennis errors is reported, but crucial details, like confidence intervals or variance in accuracy across different datasets, are missing. Just as traditional statistical analyses require detailed metrics (effect sizes, p-values), AI models must be rigorously evaluated using standard performance metrics. In example: (i) Report accuracy, precision, recall, and F1-scores for error identification; (ii) Include confidence intervals or standard deviations for these metrics to indicate the model’s robustness; (iii) Clarify whether cross-validation was used to ensure that the model is not overfitting to a particular set of data; and (iv) Include a confusion matrix to show where the model succeeds or fails in classifying various errors (e.g., racket position vs. footwork).

**Response:** We greatly appreciate your insightful suggestion. Consequently, we have included confusion matrices for each category in Figure 5.b to provide a more standardized and visual presentation of the system's performance in identifying table tennis errors. Additionally, we have expanded the content to explain the distinctions in the system's ability to differentiate various types of table tennis errors, along with potential reasons for these differences.

**Response to comments:10.**(P. 12 L. 10–20) - The article does not clarify whether the outputs generated by GPT-4 were evaluated for quality, accuracy, or consistency. This is essential, as one would expect an evaluation of any AI system’s output similar to how confidence intervals or p-values are reported in statistical studies. As a reviewer I have a few questions: (i) Was the quality of GPT-4’s recommendations assessed? Were there metrics or cross-validation techniques applied to ensure the model consistently provides correct or useful feedback?; (ii) Did the authors perform any error analysis to understand common mistakes made by the model (e.g., false positives or incorrect classifications)?; (iii) If experts evaluated the system, what were the specific criteria used to measure GPT-4’s performance?

**Response：**We sincerely appreciate your insightful comments. The discussion on the evaluation details of GPT-4's responses indeed enhances the practical significance of our AI Table Tennis Coach system.Ensuring the quality of model responses is crucial. When GPT-4 provides advice for novice table tennis players, the suggestions used for expert evaluation are derived from five synchronized responses to the same sample video. Experts will provide a comprehensive assessment after considering the consistency of these responses. We have clarified this point in the manuscript.Analyzing GPT-4's errors in recognizing mistakes is vital for understanding the limitations of the AI Table Tennis Coach model and offering subsequent improvement strategies. We have conducted a detailed analysis of the AI Table Tennis Coach system using a confusion matrix, highlighting the system's weaknesses and suggesting potential improvements in the discussion section.Regarding the system's accuracy, practicality, personalization, and comprehensibility, we have included a new table in Section 3.2 with detailed explanations of the evaluation criteria. This addition strengthens the persuasiveness of our article.Thank you once again for your valuable feedback, which has significantly contributed to the enhancement of our work.
**Response to comments:11.**(P. 12) – Table 1 should be introduced in the text before the actual table.
**Response:**This insightful comments have highlighted structural shortcomings in our manuscript. We have since revised the structure, ensuring that all tables are properly introduced, making the document more accessible and comprehensible. Thank you for your valuable feedback.
3. RESULTS
**Response to comments:1.**(P. 13) – Figure 5 should be introduced in the text prior to its appearance in the document.

**Response：**Thank you for your suggestion! I will make sure to introduce Figure 5 in the text before it appears in the document to enhance clarity and context for the readers.
**Response to comments:2.**(P. 13) – The figure 6 must appear immediately after its introduction in the text.

**Response：**Thank you for your suggestion! I will make sure to introduce Figure 5 in the text before it appears in the document to enhance clarity and context for the readers.
**Response to comments:3.**(P. 13) - The system demonstrates impressive accuracies (73% for arm-related errors and 82% for racket errors). However, these percentages are not sufficient for real-world use if aiming for higher levels of training (beyond beginner-level players).• Suggestion: Clarify that these accuracy metrics are primarily for beginners and that further refinement is needed for advanced players. Also, suggest ways to increase accuracy, such as incorporating biomechanics data or higher-resolution tracking.

**Response：**Thank you for your feedback. We will specify that these accuracy metrics primarily target beginners, and propose potential solutions to improve the system’s accuracy by incorporating biomechanical data or high-resolution tracking methods.At the end of section 3.1, we added the following sentence:These accuracy metrics are primarily designed for beginners and require further optimization for advanced players. Furthermore, in order to enhance the system's accuracy in identifying beginners, it is recommended to incorporate biomechanical data or utilize higher resolution tracking methods.
**Response to comments:4.**(P. 14) - The comparison between GPT-4, Gemini Pro, and Qwen-VL is useful, but the decision criteria for selecting GPT-4 should be elaborated.• Suggestion: Add more detailed metrics to the model comparison (e.g., computation time, resource consumption, scalability) and explain why GPT-4 was chosen over the others in those dimensions, not just for accuracy and false positives

.

**Response：**We sincerely appreciate your insightful comments. Indeed, the GPT-4V series represents one of the most versatile multimodal commercial models available. In the context of commercial models, the differences in computation time, resource consumption, and scalability are relatively minimal. We have expanded our discussion to address these aspects, with the anticipation that future work will enable the development of a localized and privatized table tennis coaching system that can rival commercial solutions. Thank you once again for your valuable feedback, which has been instrumental in refining our manuscript.
**Response to comments:5.**(P. 14) – Figure 7 should be introduced in the text prior to its appearance in the document.

**Response：**Thank you for your suggestion! I will make sure to introduce Figure 7 in the text before it appears in the document to enhance clarity and context for the readers.
**Response to comments:6.**(P. 15) – Figure 9 should be introduced in the text prior to its appearance in the document.
**Response：**Thank you for your suggestion! I will make sure to introduce Figure 9 in the text before it appears in the document to enhance clarity and context for the readers.
**4. DISCUSSION**
**Response to comments:1.**(P. 16) - The cost analysis focuses primarily on beginner-level applications, but there is no mention of scaling this system for more advanced or professional users.• Suggestion: Include a section that discusses how the system could be adapted for semi-professional or professional players, with potentially more advanced (and more expensive) hardware. This would broaden the scope of the application and make the study more impactful.

**Response：**Thank you for your valuable feedback! I appreciate the suggestion to consider scalability for advanced users. In response, I have added Section 4.4, which discusses potential adaptations of the system for semi-professional and professional applications, including the possibility of using more advanced hardware. This addition broadens the study’s relevance and impact.
**Response to comments:2.**(P. 16) - The lower accuracy in recognizing footwork errors is mentioned, but more detail should be given regarding how this will be improved in future research.• Suggestion: Propose specific technical solutions, such as integrating a multi-camera system or combining inertial measurement units (IMUs) with visual data. Without a clear plan, it may appear that this limitation will remain unaddressed.

### **Response：**Thank you for highlighting this point! I agree that providing specific solutions would strengthen the discussion on improving accuracy in recognizing footwork errors. I have incorporated this suggestion into the final paragraph of the discussion, outlining a plan for future improvements. 5. CONCLUSION **Response to comments:1.**(Page 17) - The conclusion touches on future improvements in footwork recognition and training for high-level athletes, but these should be expanded with more concrete plans. **Response**：Thank you for your feedback! I have expanded the conclusion to include more concrete plans for future improvements in footwork recognition and training for high-level athletes, providing clearer direction for the system's development. 6. REFERENCES **Response to comments:1.**In most of the references, the symbol ‘[J]’ appears. Please confirm whether this is intentional.

### **Response：**I have checked and confirmed that the "[J]" symbol in the references is unnecessary and have removed it. To ensure accuracy, I have also included the page numbers where applicable.I used the PLOS ONE reference format available in Zotero for the corrections. These changes were made after carefully considering the citation format requirements.Once again, I sincerely appreciate your constructive feedback. I have made all the necessary revisions to ensure the manuscript meets the highest quality standards.Thank you for your valuable suggestions regarding our manuscript. I have carefully reviewed and revised the reference section to ensure it adheres to the required format.

**Response to Reviewer 2**

**Response to comments: 1. There is a gap between highlighting the novel contributions as the approaches have been utilized for the study. Can the authors explicitly discuss and highlight the novelty of the paper? As the authors employed existing approaches.**

**Response:** Thank you for your insightful comments on our methodology. We acknowledge the use of established approaches in this work; however, the application of a multimodal large language model (LLM) based AI coaching system in the domain of table tennis remains unprecedented. Our workflow is particularly tailored to beginner-level table tennis instruction and, under our framework, demonstrates advantages over directly using a base LLM for training assistance.

**Response to comments: 2. Please conduct analysis and provide the ANOVA/Cohen's kappa scores based on the experts’ rating. The authors envision employing the proposed TT coaching system to enhance training efficiency; if so, can the authors explicitly show the improvement rate before and after the training session? We encourage the authors to release the data and code during the review process.**

**Response:** We completely agree with the reviewer’s suggestion. To address this, we have added a detailed section (Section 3.2) that includes an analysis of expert ratings. Training in sports is inherently complex and requires collaboration between both coaches and athletes. While the TT coaching system significantly reduces beginners' dependency on direct coaching, we acknowledge that real coaching remains indispensable. We believe that with the assistance of LLMs, table tennis can become more accessible to a broader audience. The data and code for our system have been made publicly available on GitHub at [https://github.com/mwlsus/ttcs_by_llm](https://github.com/mwlsus/ttcs_by_llm).

**Response to comments: 3. There is a lack of related literature comparison as to where this work fits and outperforms SOTA methods. - Please provide a tabular comparison.**

**Response:** This comments have been highly enlightening. While AI-based table tennis instruction is an emerging area, existing works have primarily focused on tasks such as intelligent refereeing and trajectory tracking. Our focus, however, is on offering user-friendly guidance specifically for beginners, where, to our knowledge, no stable benchmark exists. In response, we have added a comparative description of related works in the introduction section.

**Response to comments: 4. Please provide an ablation study or rationale for adopting the proposed pipeline. We found this part of the motivation is lacking throughout the paper.**

**Response:** We share the reviewer’s interest in this point. Multimodal LLMs in sports technology are advancing rapidly, with new methodologies achieving increasingly promising outcomes. Our TT coaching system emphasizes beginner-level table tennis instruction, specifically in error recognition, showing improved outcomes over directly applying a baseline LLM for training. We have added further elaboration on this in Section 3.1.

**Response to comments: 5. Discuss the proposed framework's limitations, particularly scalability and real-world deployment, to provide a more balanced perspective on its applicability.**

**Response:** We agree that discussing scalability and real-world deployment is essential. Our AI coaching model has been designed to be as low-cost and deployable as possible; however, using commercial models still poses cost challenges, and current limitations mean it requires a certain level of technical proficiency from users, falling short of full automation. We have expanded on these limitations in Section 4 as part of our future development goals.

**Response to comments: 6. Please provide the feasibility test on how the authors plan to deploy in resource-constrained devices.**

**Response:** The reviewer’s point regarding deployment in resource-constrained environments is very pertinent. Currently, our table tennis coaching system relies on commercial LLM interfaces, with the most intensive processing handled in the cloud. However, deploying real-time body motion detection on limited-resource devices remains a challenge. Moving forward, we aim to explore deployment on edge devices with quantized open-source multimodal LLMs, such as Qwen-VL or InternVL, enabling a personal pocket-sized table tennis coach. We have added more discussion on this point in Section 4.

**Response to Reviewer 3**

1. **Reviewer Comment:**

The authors state that data and code are publicly available without restrictions. However, no direct links to the code and data are provided. Including an anonymous GitHub repository would provide more details about the data and code framework for reproduction purposes and help people to understand the framework.

**Response:**

Thank you for your valuable feedback. We have made the data and code publicly available on our GitHub repository at: [https://github.com/mwlsus/ttcs_by_llm](https://github.com/mwlsus/ttcs_by_llm).

2. **Reviewer Comment:**

The authors mention a knowledge base that provides the theoretical foundation for MLLMs. It would be beneficial to clarify whether this knowledge base is text-based or video-based. A more detailed explanation or an example would help illustrate this clearly.

**esponse:**

We appreciate your interest in the knowledge base used in our study. Currently, we utilize a text-based knowledge base, which encompasses most of the relevant information in a question-and-answer format, including common beginner mistakes, error patterns, and correct postures. While a video-based knowledge base could potentially yield better results, we have not yet identified an effective method for video data matching. We have provided a more detailed explanation in Section 2.5 of the revised manuscript.

3. **Reviewer Comment:**

It would be valuable to compare the performance of state-of-the-art MLLMs on this task. A comparison between the authors' model and SOTA MLLMs would help clarify the contribution of this work. The paper lacks a clear explanation of how visual recognition, motion capture, and large language models are integrated. How is the video data incorporated into the MLLM models? The authors use pre-trained recognition algorithms to identify players and ball trajectories, but the advantages of this approach are not clearly stated. Does this perform better than directly uploading the video to the MLLMs? A detailed results comparison would help support the authors' claims.

**Response:**

Thank you very much for your insightful comments. There has been considerable work on AI teaching for table tennis, including intelligent refereeing and trajectory recognition. However, our work focuses on providing accessible guidance for beginners. Currently, there is no stable baseline in the domain of table tennis instruction for novices, which remains our ongoing goal. Specifically, for beginner table tennis instruction, particularly in error recognition, our approach has shown superiority over directly utilizing raw large language models for auxiliary training. We have added a description of this point in Section 3.1 of the revised manuscript.

Additionally, we acknowledge that many existing large models can directly accept video input. However, current multimodal large language models do not yet perfectly support video processing; most video-level multimodal models perform frame-by-frame recognition, which introduces more noise. Our approach leverages pre-trained recognition algorithms to identify key frames and motion trajectories, providing more meaningful information. Consequently, our AI table tennis coaching system directly inputs key frames and action summary charts into the multimodal large language model, aiming for better performance. We have supplemented this explanation in the new Section 2.5.

4. **Reviewer Comment:**

In Table 1, the meaning of "Proportion" and "Quantity" is unclear. Does this represent the test subset or the full dataset proposed by the authors? The proportions do not sum to 100%. A better clarification of the dataset would help readers understand the size of the dataset and the proportion used as the test set.

**Response:**

We appreciate the reviewer's valuable suggestion and have made the corresponding modifications to improve clarity. The proportions in Table 1 may sum to more than 100% because table tennis errors can result from multiple combined mistakes. To enhance the robustness of the dataset, we have included information on annotation consistency for labeling errors in Table 1. Additionally, we have provided a detailed description of the expert annotation process in Section 2.4 of the revised manuscript.

5. **Reviewer Comment:**

In Figure 7, the authors claim "Automated loss analysis, error cause investigation, and recommendations for a table tennis novice in an 11-point match." The AI-generated suggestions seem applicable to most people who make such errors. There is a concern that the generated suggestion content is based on error type rather than personalized movement videos. Is it possible to prompt the model to show different arm angles where the player makes mistakes, since Figure 3 suggests arm angles can be captured? This would make the model's output appear more personalized.

**Response:**

Thank you for your insightful suggestion. Enabling the AI table tennis coach to provide responses enriched with both text and images is indeed highly valuable. We can specify in the AI coach's prompt to include relevant images and require corresponding explanations. Such responses would facilitate better guidance for players. Implementing the rendering of returned content would also require advanced web front-end techniques, which we plan to address in our future development efforts.
